# Associations between individual and environmental factors and adolescent substance use in the border regions of the Netherlands, Belgium, and Germany

**DOI:** 10.3389/fpubh.2026.1858516

**Published:** 2026-09-08

**Authors:** Brigitte A. M. van der Zanden, Stephanie Wagner, Christian J. P. A. Hoebe

**Affiliations:** 1 Department of Social Medicine, Care and Public Health Research Institute (CAPHRI), Faculty of Health, Medicine and Life Sciences, Maastricht University, Maastricht, Netherlands; 2 WHO Collaborating Centre Regions for Health and Cross Border Public Health, Maastricht University, Maastricht, Netherlands; 3 Department of Knowledge and Innovation, South Limburg Public Health Service, Heerlen, Netherlands; 4 Department of Sexual Health, Infectious Diseases and Environmental Health, Living Lab Public Health Mosa, South Limburg Public Health Service, Heerlen, Netherlands; 5 Department of Medical Microbiology, Infectious Diseases and Infection Prevention, Care and Public Health Research Institute (CAPHRI), Faculty of Health, Medicine and Life Sciences, Maastricht University Medical Centre (MUMC+), Maastricht, Netherlands

**Keywords:** age-friendly policy, border region, cross-border collaboration, cross-border policy exchange, healthy aging, local government, Meuse-Rhine Euroregion, municipal policy

## Abstract

**Introduction:**

Adolescent use of alcohol, tobacco, and cannabis remains a significant public health concern across Europe. In border regions, young people may be exposed to differing laws, social norms, and environmental influences, creating a context in which individual and environmental associations may operate differently. This study investigates how individual and environmental factors relate to substance use among adolescents in the border regions of the Netherlands, Belgium, and Germany.

**Methods:**

The study included 20,461 adolescents (aged 13–17) from the cross-border regions of the Netherlands, Belgium, and Germany. In 2023, they completed a standardized, anonymous online questionnaire during school hours. The questionnaire assessed substance use (alcohol, tobacco, cannabis), psychosocial characteristics, and physical, social, and economic environmental factors and was analysed using descriptive statistics and logistic regression.

**Results:**

Higher odds of binge drinking, smoking and vaping, and cannabis use were observed for older age, peer influence, and frequent late-night outings, while lower odds were observed for parental monitoring, clear household rules, and structured leisure. While the strength of these associations varied across countries, the underlying factors were largely consistent.

**Conclusion:**

Behavioral differences between countries may reflect variations in national policies and cultural contexts, as well as the broader social dynamics that characterize life in cross-border regions. The social and cultural environments in which adolescents grow up appear to be more decisive. Effective prevention in border regions should therefore prioritize context-specific, cross-border collaboration, focusing on strengthening social structures around parents, schools, and leisure time.

## Introduction

Unhealthy lifestyle behaviors among adolescents continue to pose significant challenges across many European member states, particularly in relation to substance use, including alcohol, cannabis, and tobacco. Findings from the World Health Organization’s Health Behavior in School-aged Children (HBSC) survey indicate that 57% of 15-year-olds across Europe, Central Asia, and North America have experimented with alcohol, and 40% consumed it within the past 30 days (1, 2). Although the HBSC has shown alcohol use among adolescents has declined in the recent years (1, 2), early initiation remains concerning due to its potential consequences for brain development, risk of addiction, and risky behaviors.

Similar risks are associated with early cannabis and tobacco use. Early cannabis use has been linked to cognitive impairments, structural brain changes and lower academic achievement (3). Adolescent tobacco use is strongly associated with nicotine dependence (4), long-term respiratory and cardiovascular problems (5), and is associated with an increased likelihood of progressing to poly-substance use (6), although this relationship is debated and not consistently supported across studies (7, 8). When initiated at a young age, these substances, like alcohol, can have compounding negative effects, highlighting the importance of early prevention efforts.

According to the same HBSC survey, 6% of 15-year-olds reported using cannabis in the past 30 days, indicating recent rather than frequent use. While traditional cigarette smoking is decreasing, the use of e-cigarettes is on the rise: 32% of 15-year-olds have tried them, and 20% reported recent use (1, 2).

Research consistently demonstrates that adolescent substance use is a complex issue involving a combination of health, developmental, and societal risks. Substance use can significantly harm both physical and mental health by disrupting normal brain development, increasing the risk of future addiction, and contributing to various behavioral problems. Addressing these risks requires developing effective strategies, activities, and interventions that foster a healthy living environment, with a focus on key areas including individual and environmental factors, such as demographics, family situation, peer groups, school structure, and leisure activities. Such an approach is reflected in prevention frameworks used in the three countries, including Dutch initiatives inspired by the Icelandic Prevention Model (IPM) (9, 10), Communities That Care (CTC) in Germany (11, 12), and Belgian school- and environment-based prevention frameworks inspired by the European Drug Prevention Quality Standards (EDPQS) (13) and the COMIQS.BE consensus project (14).

Border regions within the European Union represent complex socio-spatial environments where daily life often transcends national boundaries. Mobility for schooling, leisure, and work creates shared socio-economic spaces shaped by both border permeability and the degree of administrative coordination between neighboring states (15–17). Scholars in border studies describe these areas as dynamic contact zones influenced by both integration and division, where the meaning of ‘borderlessness’ remains uneven and context-dependent (15–17). While the EU promotes cross-border cooperation in health and social policy, competencies for prevention and health promotion remain primarily national or even regional (18). Recent analyses also suggest that a genuinely European mindset in public health policymaking is still lacking (18). This tension between European integration and nationally anchored systems makes border areas particularly relevant for studying how individual and environmental determinants of adolescent substance use operate within differing legal, cultural, and institutional contexts.

Within this broader European context, national regulations on adolescent substance use differ across the three countries. For alcohol, the minimum age is 18 years in the Netherlands, whereas in Germany and Belgium beer and wine may legally be purchased from age 16 (spirits from 18). In Germany, supervised consumption of beer or wine is permitted from age 14 when accompanied by a custodial parent. For tobacco and e-cigarettes, sales to minors (<18 years) are prohibited in all three countries. Regarding cannabis, possession and purchase are prohibited for minors across all countries. The Dutch tolerance policy (‘coffeeshops’) applies only to adults (≥18), and Germany’s partial adult-use legalization took effect only in 2024. These differences shape the legal environment encountered by adolescents in border regions and provide context for interpreting cross-national patterns.

Together, these three approaches converge on the principle that resilience is strengthened by positive factors like supportive social networks and stable family environments, while risks are heightened by negative influences like poverty, and weak parental monitoring. This convergence provides the rationale for the inclusion of both individual and environmental variables in the present study, which is also supported by extensive evidence reviews on risk and protective factors for adolescent substance use (19, 20).

Despite the existence of these three frameworks, research remains limited in certain contexts, particularly in border regions. These border regions may present social and structural conditions that are relevant to substance use but have not yet been thoroughly studied. Border regions are characterized by economic, social, cultural, and political dynamics related to their proximity to national boundaries. At the same time, border regions often face specific challenges, like economic disparities, language differences, and regulatory variations, but they also benefit from cross-border cooperation opportunities that can drive regional development and integration. This research gap is especially urgent given that about one-third of European citizens reside in border regions (21–23), including about 28% of adolescents aged 10 to 19 (21). Citizens, including adolescents, cross borders for shopping, education, work, or leisure (24–26), subjecting them to varying laws and cultural influences related to alcohol and tobacco, which may shape their choices and behaviors. Moreover, in cross-border regions, differences in taxation and pricing of alcohol and tobacco can influence their accessibility, making these substances more or less available to adolescents and potentially impacting consumption patterns. While many policy strategies emphasize creating safe, healthy, and supportive environments for adolescents, the geographical context of border regions remains understudied (15–17). This study therefore focuses on adolescents living in the border regions of the Netherlands, Belgium and Germany, and presents a large-scale comparison of substance use and social determinants in these border areas.

These considerations highlight the need to examine how individual and environmental factors are associated with adolescent risk behavior in cross-border settings. Differences in regulations and cultural norms across neighboring countries may influence adolescent substance use. A deeper understanding of these influences is essential for developing effective, evidence-based interventions and policies tailored to the characteristics to border regions. Therefore, there is a need to examine how individual and environmental factors associate with substance use vary across border regions and how these patterns relate to alcohol consumption, tobacco smoking and vaping, and cannabis use. By analysing patterns of binge drinking, smoking or vaping, and cannabis use in border regions of the Netherlands, Germany, and Belgium, this study leverages the cross-border setting to explore both similarities and differences in adolescent behavior. The insights gained will inform the development of targeted prevention strategies and policies to mitigate health risks associated with substance use among adolescents in border regions (10, 19, 20, 27–31).

## Methods

### Study design and study population

The Youth Euregional Scan (YES) was a cross-sectional survey study among 13-17-year-olds in school-setting in border regions of the Netherlands, Germany, and Belgium. These border regions are part of the so-called Meuse-Rhine and Rhine-Meuse North Euroregions, encompassing several municipalities that vary in size and character, ranging from rural communities to urban centers, that are geographically close but administratively belong to different countries. In September 2023, data collection was conducted in secondary schools within these regions using a standardized online questionnaire. Collected information included data on substance use, along with individual and environmental determinants influencing these behaviors, such as age, gender, parental supervision, and social support. Recruitment procedures for schools, by convenient sampling, differed by country. In the Netherlands, participation was coordinated by the regional public health services (GGD) as part of their statutory youth health survey, which is conducted every 4 years and has high school-level participation. In Germany, most schools were approached for the first time through local public health authorities, while a few schools had participated in earlier cross-border youth surveys, the survey is not structurally embedded in the tasks of health authorities. In Belgium, schools were approached through the national public health authority, which applied a sampling procedure for schools, but participation was voluntary and took place in a context where schools are frequently invited to other surveys. All secondary schools in the border regions were invited to participate, ensuring equal opportunity for inclusion, resulting in a convenient sample. Because all eligible secondary schools within the border regions were invited to participate through the respective regional public health authorities, formal participation rates at the school level were not calculated. The analyses were conducted at the individual level, based on all adolescents who completed the full questionnaire. Participation was voluntary, the final sample size depended on the number of schools and students that responded. While not all schools participated, the study included 100 schools located in the border region, representing various educational school levels. Only second- and fourth-grade students at secondary school, focusing on the 14- and 16-year-olds, were invited to participate. Because entire classes were included, the actual age range of participants was 13–17 years. Analyses were therefore restricted to adolescents in this age range. Participation was voluntary and both students and their parents could decline participation or withdraw at any time. The questionnaire was administered online via a secure digital platform during regular school hours under classroom supervision. Data collection took place in a standardized manner, with students completing the survey individually using school computers or tablets under the guidance of a teacher or school staff member. This controlled setting ensured comparable conditions across schools and minimized potential response bias. The survey was conducted anonymously, as no personal identifying information, such as names, dates of birth, or addresses, was collected. Residential address data and information on individual cross-border mobility were not collected due to privacy and feasibility reasons. As a result, analyses are based on school location within border regions rather than on participants’ distance to the border or frequency of cross-border movements. Only participants with no missing data on either dependent or independent variables were included in analyses.

Questionnaires were included in the analyses only if they were fully completed. Respondents who left one or more survey items unanswered were excluded prior to analysis, regardless of which variable was missing. Therefore, the analytic sample consisted exclusively of complete cases, and no item-level missing data were present in the dataset. This approach ensured that the descriptive statistics and regression analyses were based on identical respondent groups across all variables.

### Measurements

Outcome variables included alcohol, smoking and vaping, and drug use. Alcohol-related outcomes included self-reported alcohol consumption: been drunk or tipsy in the past 4 weeks (never, 1 time, 2 times, 3 times, 4–10 times, ≥11 times), and binge drinking (≥5 drinks on one occasion) in the past 4 weeks (never, 1 time, 2 times, 3–4 times, 5–6 times, 7–8 times, ≥9 times; dichotomizes into yes/no). Access to alcohol was assessed through whether participants who ever drank alcohol purchased it themselves (yes/no), or received alcohol from others (yes/no), more specifically from parents/guardians, other adults, or peers/acquaintances (yes/no). Perceived parental attitudes toward alcohol use were also measured (approve, think child should drink less alcohol, advise against, forbid, say nothing about, do not know).

Smoking and vaping now (daily, at least weekly, current) were assessed and analysed both separately and combined (yes/no). Cannabis use was assessed for both lifetime (yes/no) and past 4-week prevalence (yes/no). Use of other substances (e.g., ecstasy, cocaine, magic mushrooms, nitrous oxide) was assessed similarly, both lifetime (yes/no) and within the past 4 weeks (yes/no).

The independent variables included questions on key variables of substance use, based on evidence identified by previous studies on substance use and were related to individual factors and environmental factors. The selection of independent variables was guided by established prevention frameworks (IPM, CTC, EDPQS/COMIQS) and by evidence reviews identifying key risk and protective factors for adolescent substance use (19, 20, 32). Accordingly, the questionnaire included both individual-level determinants (e.g., demographics, psychological well-being, school experiences) and environmental-level determinants (e.g., parental monitoring, peer behavior, leisure activities). The available items in the survey largely reflected these domains, allowing us to examine both individual and contextual influences in a cross-border setting.

Individual factors included demographic variables such as gender (female vs. male; other excluded for analysis) and school grade (2nd vs. 4th). Psychosocial factors encompassed psychological well-being (total score on the 5-item Mental Health Inventory, MHI-5), feeling stressed by one or more of the following factors: school or homework, home situation, own problems, what others think of them, everything they have to do (often/very often = yes, never/almost never, sometimes = no), and regularly/often perceived performance pressure from themselves or others, including pressure from parents, teachers, friends, and coaches (yes/no). Other variables included the possibility to turn to someone when having a problem or feeling troubled (yes/no), bullied at school in the last 3 months (yes/no), perceiving school as pointless (agree/strongly agree = yes, completely disagree, disagree, neither agree nor disagree = no), and school experience ((really) like, okay, not like/terrible).

Environmental factors covered both the physical and social environment, as well as the economic environment. Physical environment variables included being out of the home after 22:00 for three or more days in the past week (yes/no), and engagement in extracurricular activities in a club or organisation as music or arts, organized sports (sport club or gym), and a different kind of organization, as well as physical activity without a club ((almost) never, less than 1 day per week, 1 day per week, 2 or 3 days per week, 4 to 6 days per week, every day). Social environmental variables included peer alcohol use (most/(almost) all friends = yes, none/some = no), as well as parents/guardians usually know whereabouts, clear rules from parents, and time parents and child spent together (completely agree/agree = yes, completely disagree, disagree, neither agree nor disagree = no). Economic environment was assessed through perceived difficulty managing finances at home (some/significant difficulty = yes, no difficulty/no difficulty but need to be cautious = no).

### Source and comparability

The questionnaire was administered in a standardized manner across all regions, ensuring consistency in the items, response options, and timing of data collection for all participants, making it irrespective of regional differences in culture or regulatory frameworks. The use of a uniform digital platform further enabled reliable comparisons across geographical and socio-economic subgroups within this cross-border context. Anonymous data collection via unique access codes helped mitigate social desirability and interviewer bias, while the inclusion of diverse school types and both urban and rural regions improved representativeness. Additionally, careful translation and cultural adaptation was performed by the research team to minimize interpretation bias. Overall, these safeguards contribute to the validity of the findings in this cross-border study.

### Statistical method

Descriptive analyses were performed to examine the characteristics of the study population. To assess the differences between participants from the Netherlands, Germany and Belgium, we performed chi-square tests with Bonferroni adjusted post hoc tests. For each substance use outcome separately, an empty multilevel logistic regression model was estimated with adolescents nested within regions and country as fixed effect. Descriptive Intraclass Correlation Coefficients (ICC) were estimated as an indication of clustering.

We conducted multi-variable logistic regression analyses to examine the association of the individual and environmental factors with substance use, stratified for country, calculating odds ratios (OR) with 95% confidence intervals. A priory, collinearity between independent variables was checked using Pearson correlation coefficients. All correlations were below 0.60 suggesting that multicollinearity was not a major concern (see Supplementary Table S1). In addition, variance inflation factors (VIF) were examined, with all values ranging between 1.07 and 1.90, confirming the absence of problematic multicollinearity. Detailed VIF results are provided in Supplementary Table S2. All analyses were conducted using IBM SPSS software version 27.0 (IBM Corp. Armonk, NY, USA). A *p*-value <0.05 was considered statistically significant.

## Results

**Figure 1:**
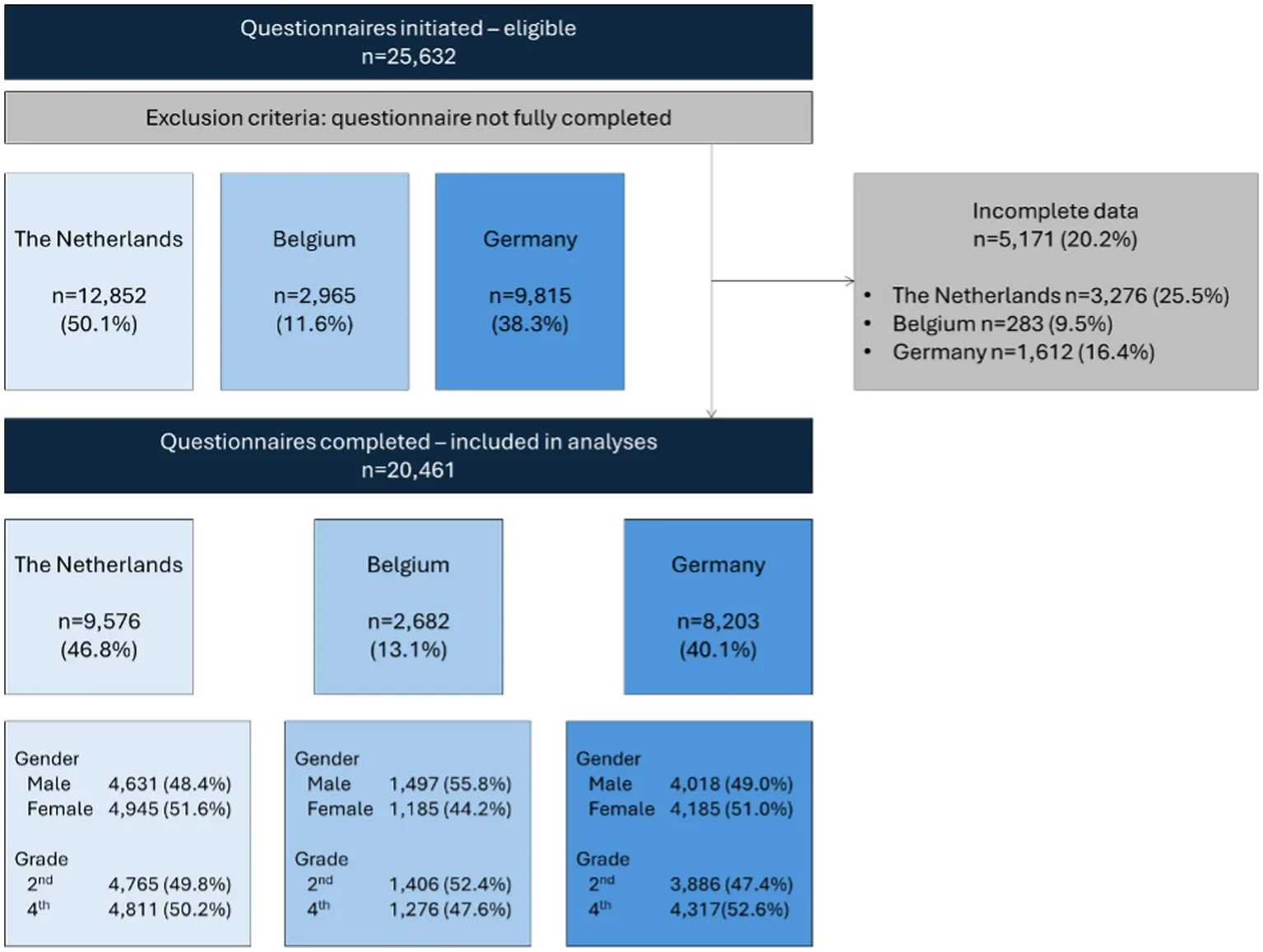
Flowchart of questionnaire participation and inclusion across the Netherlands, Belgium, and Germany, including reasons for exclusion and final analytic sample by gender and grade level.

A total of 25,632 adolescents participated in the study, of whom 20,461 completed the full questionnaire. The remaining 5,171 incomplete questionnaires were excluded from analysis. A non-response analysis comparing included and excluded respondents on available demographic characteristics suggests that selective non-response was limited. Figure 1 provides a visual overview of the participant flow, including initial participation, exclusion due to incomplete data, and the final analytic sample stratified by country, gender, and grade level.

The included sample was broadly balanced by gender in the Dutch (48.4% male) and German (49.0% male) border regions, while in the Belgian border region boys were slightly overrepresented (56%). Grade distributions were comparable across countries, with approximately equal proportions of adolescents in the 2nd (13–14 years) and 4th (15–16 years) grades (Figure 1). To assess the extent of clustering at the regional level, empty multilevel logistic regression models were estimated for each outcome, with children nested within regions and country included as fixed effect. The intraclass correlation coefficients (ICCs), were low across outcomes (alcohol use = 0.03, smoking and vaping = 0.06, and cannabis use = 0.02), indicating limited clustering at the regional level. Given that the regional units were broad and internally heterogeneous, and that the main explanatory variables were measured at the individual and immediate social or environmental level, standard logistic regression models were used for the main analyses.

In the following sections, results are presented by substance (alcohol, smoking/vaping, and cannabis), combining descriptive and regression findings for each outcome. Tables 1–3 summarize the descriptive characteristics of substance use by country, whereas Tables 4 and 5 present the statistically significant factors associated with higher and lower odds of substance use derived from the multivariable regression analyses. A summary of individual and environmental characteristics that provides broader context for interpreting these observed behavioral patterns is presented at the end (Table 6). Full regression outputs, including all odds ratios (OR) and 95% confidence intervals (CIs), are provided (Appendix I).

**Table 1:** Overview of individual and environmental factors in the three countries.

Item	Answer	Netherlands			Belgium			Germany		
		*n*=	%	Group diff.	*n*=	%	Group diff.	*n*=	%	Group diff.
Gender in two categories***	Boy	4,631	48.4%	a	1,497	55.8%	b	4,018	49.0%	a
	Girl	4,945	51.6%	a	1,185	44.2%	b	4,185	51.0%	a
Class***	2nd grade	4,765	49.8%	a	1,406	52.4%	b	3,886	47.4%	c
	4th grade	4,811	50.2%	a	1,276	47.6%	b	4,317	52.6%	c
Feels (very) often stressed by one or more of the following factors: school or homework, home situation, own problems, what others think of them, everything they have to do***	Yes	4,900	51.2%	a	1,514	56.5%	b	5,217	63.6%	c
Can turn to someone when having a problem or feeling troubled***	Yes	9,124	95.3%	a	2,457	91.6%	b	7,299	89.0%	c
Experiences performance pressure regularly/often (from themselves or others) ***	Yes	3,505	36.6%	a	957	35.7%	a	3,376	41.2%	b
Experiences performance pressure regularly/often, including pressure from parent(s)/guardian(s) ***	Yes	1,337	14.0%	a	406	15.1%	a	1,418	17.3%	b
Experiences performance pressure regularly/often, including pressure from teachers/school***	Yes	1,096	11.4%	a	324	12.1%	a	1,282	15.6%	b
Experiences performance pressure regularly/often, including pressure from friends***	Yes	759	7.9%	a	229	8.5%	a	883	10.8%	b
Experiences performance pressure regularly/often, including pressure from coach or trainer in sports***	Yes	403	4.2%	a	147	5.5%	b	447	5.4%	b
Bullied at school in the last 3 months***	Yes	1,331	13.9%	a	390	14.5%	a	2,202	26.8%	b
Finds going to school pointless***	Yes	2,038	21.3%	a	483	18.0%	b	1,224	14.9%	c
School experience in 3 categories***	(Really) like	3,259	34.0%	a	1,134	42.3%	b	3,547	43.2%	b
	Okay	4,466	46.6%	a	1,076	40.1%	b	2,987	36.4%	c
	Not like/ terrible	1,851	19.3%	a	472	17.6%	b	1,669	20.3%	a
Child is not at home for 3 days or more after 22:00***	Yes	1,675	17.5%	a	343	12.8%	b	1,302	15.9%	c
On how many days of the week do you engage in music, singing, acting, drawing/painting, or photography outside school hours with a club or association?	(Almost) never	5,890	61.5%		1,567	58.4%		4,726	57.6%	
	Less than 1 day per week	684	7.1%		172	6.4%		520	6.3%	
	1 day per week	910	9.5%		246	9.2%		1,091	13.3%	
	2 or 3 days per week	1,084	11.3%		389	14.5%		1,158	14.1%	
	4 to 6 days per week	431	4.5%		130	4.8%		386	4.7%	

**Table 2:** Overview of alcohol use in the three countries.

Item	Answer	Netherlands			Belgium			Germany		
		*n*=	%	Group diff.	*n*=	%	Group diff.	*n*=	%	Group diff.
In the last 4 weeks, has drunk alcohol	Yes	3,149	32.9%	a	943	35.2%	a	2,793	34.1%	a
In the last 4 weeks, been drunk or tipsy	Yes	1,691	17.7%	a	485	18.2%	a	1,398	17.1%	a
In the last 4 weeks, 5 or more drinks on 1 occasion (binge drinking)	Yes	2,076	21.7%	a,b	614	22.9%	b	1,695	20.7%	a
I receive alcohol from other adults (full glass or more)***	Yes	926	26.8%	a	186	18.8%	b	560	18.7%	b
I receive alcohol from my parents/guardians (full glass or more)***	Yes	1,701	49.3%	a	417	42.1%	b	1,382	46.3%	b
I receive alcohol from a friend or acquaintance (full glass or more)***	Yes	1,671	48.4%	a	395	39.9%	b	1,478	49.5%	a
I let others buy alcohol (a friend, acquaintance, or stranger) (full glass or more)	Yes	1,043	30.2%	a	286	28.9%	a	945	31.6%	a
I buy alcohol MYSELF (full glass or more)***	Yes	593	17.2%	a	318	32.1%	b	809	27.1%	c
Opinion parents alcohol use (full glass or more)***	They approve	1,369	38.4%	a	386	37.8%	a	1,516	49.2%	b
	They think I should drink less alcohol	260	7.3%	a	69	6.8%	a	243	7.9%	a
	They advise against it	901	25.3%	a	230	22.5%	a	545	17.7%	b
	They forbid it	126	3.5%	a	33	3.2%	a	131	4.3%	a
	They say nothing about it	573	16.1%	a	200	19.6%	b	256	8.3%	c
	They do not know	338	9.5%	a	103	10.1%	a,b	389	12.6%	b
Some to (almost) all friends drink alcohol***	Yes	6,486	67.7%	a	1,719	64.1%	b	4,954	60.4%	c

Values are absolute numbers (*n*) and percentages (%), calculated on complete cases per country (Netherlands *n* = 9,576; Belgium *n* = 2,682; Germany *n* = 8,203). Only affirmative (“Yes”) responses are shown for dichotomous items; the remaining percentages represent “No” responses. For multi-category items, all relevant answer categories are presented. Asterisks (*, **, ***) indicate statistically significant overall between-country differences based on chi^2^ tests (*p < 0.05, **p < 0.01, ***p < 0.001). Superscripts a, b, c within a row denote significant pairwise differences between countries according to Bonferroni-adjusted post-hoc comparisons (α = 0.05); identical letters indicate no significant difference.

**Table 3:** Overview of smoking and vaping in the three countries.

Item	Answer	Netherlands			Belgium			Germany		
		*n*=	%	Group diff.	*n*=	%	Group diff.	*n*=	%	Group diff.
Currently smokes daily***	Yes	260	2.7%	a	98	3.7%	b	509	6.2%	c
Currently smokes ≥ weekly***	Yes	450	4.7%	a	147	5.5%	a	775	9.4%	b
Currently smokes every day/every week/less than once a week***	Yes	718	7.5%	a	236	8.8%	a	1,135	13.8%	b
Currently vapes daily***	Yes	610	6.4%	a	117	4.4%	b	544	6.6%	a
Currently vapes ≥ weekly***	Yes	1,004	10.5%	a	210	7.8%	b	927	11.3%	a
Currently smokes or vapes daily***	Yes	696	7.3%	a	170	6.3%	a	707	8.6%	b
Currently smokes or vapes ≥ weekly***	Yes	1,108	11.6%	a	264	9.8%	b	1,070	13.0%	c

Values are absolute numbers (*n*) and percentages (%), calculated on complete cases per country (Netherlands *n* = 9,576; Belgium *n* = 2,682; Germany *n* = 8,203). Only affirmative (“Yes”) responses are shown for dichotomous items; the remaining percentages represent “No” responses. For multi-category items, all relevant answer categories are presented. Asterisks (*, **, ***) indicate statistically significant overall between-country differences based on chi^2^ tests (*p < 0.05, **p < 0.01, ***p < 0.001). Superscripts a, b, c within a row denote significant pairwise differences between countries according to Bonferroni-adjusted post-hoc comparisons (α = 0.05); identical letters indicate no significant difference.

**Table 4:** Associations of individual and environmental factors with adolescent substance use – factors associated with higher odds.

Factors associated with higher odds of substance use				
Substance use	Nr.	Netherlands (odds)	Belgium (odds)	Germany (odds)
Binge drinking	1.	Most to (almost) all friends drink alcohol (9.56)***	Most to (almost) all friends drink alcohol (11.23)***	Most to (almost) all friends drink alcohol (9.11)***
	2.	Child is not at home for 3 days or more after 22:00 (2.95)***	Child is not at home for 3 days or more after 22:00 (3.43)***	Being in 4th grade (3.04)***
	3.	Being in 4th grade (2.62)***	Being in 4th grade (2.26)***	Child is not at home for 3 days or more after 22:00 (2.62)***
	4.	Finds going to school pointless (1.46)***	Participate in sports at a club or gym: 2–3 days per week (1.82)**	Can turn to someone when having a problem or feeling troubled (1.42)*
	5.	Participate in sports at a club or gym: 2–3 days per week (1.36)**	Can turn to someone when having a problem or feeling troubled (1.59)*	Participate in sports at a club or gym: 1 day per week (1.38)* 2–3 days per week (1.39)** 4–6 days per week (1.37)*
Smoking/Vaping	1.	Most to (almost) all friends drink alcohol (4.39)***	Most to (almost) all friends drink alcohol (7.55)***	Most to (almost) all friends drink alcohol (3.90)***
	2.	Child is not at home for 3 days or more after 22:00 (3.05)***	Child is not at home for 3 days or more after 22:00 (4.45)***	Child is not at home for 3 days or more after 22:00 (3.15)***
	3.	Finds going to school pointless (1.77)***	Experience some or significant difficulty managing finances at home (1.98)*	Experiences performance pressure from parent(s)/guardian(s) (1.54)**
	4.	Feels (very) often stressed by one or more of the following factors: school or homework, home situation, own problems, what others think of them, everything they have to do (1.43)***	Finds going to school pointless (1.50)*	Experience some or significant difficulty managing finances at home (1.42)**
	5.	Being in 4th grade (1.41)***	-	Finds going to school pointless (1.30)*
Cannabis use	1.	Most to (almost) all friends drink alcohol (4.55)***	Most to (almost) all friends drink alcohol (6.45)***	Most to (almost) all friends drink alcohol (5.31)***
	2.	Child is not at home for 3 days or more after 22:00 (3.63)***	Child is not at home for 3 days or more after 22:00 (3.29)***	Child is not at home for 3 days or more after 22:00 (3.20)***
	3.	Being in 4th grade (2.45)***	Being in 4th grade (1.89)*	Finds going to school pointless (1.45)**
	4.	Feels (very) often stressed by one or more of the following factors: school or homework, home situation, own problems, what others think of them, everything they have to do (1.41)*	–	–
	5.	Finds going to school pointless (1.35)*	–	–

Odds ratios (OR) with 95% confidence intervals (CI) were obtained from multivariable logistic regression analyses stratified by country. The table presents all statistically significant factors associated with higher odds of adolescent substance use. Complete regression outputs are provided in Appendix I. *p < 0.05, **p < 0.01, ***p < 0.001.

**Table 5:** Associations of individual and environmental factors with adolescent substance use—factors associated with lower odds.

Factors associated with lower odds of substance use				
Substance use	Nr.	Netherlands (odds)	Belgium (odds)	Germany (odds)
Binge drinking	1.	Parents/guardians usually/always know where he/she is when not at home (0.57)***	Parents/guardians usually/always know where he/she is when not at home (0.58)**	Parents/guardians have clear rules about what the child can and cannot do (0.72)***
	2.	Sport or move in free time without a club or gym: 4–6 days per week (0.64)** Every day (0.71)**	Being a girl (0.67)**	Parents/guardians usually/always know where he/she is when not at home (0.76)*
	3.	Engaging in music, singing, acting, drawing/painting, or photography outside school hours with a club or association: 2–3 days per week (0.76)*	Parents/guardians have clear rules about what the child can and cannot do (0.75)*	Experiences performance pressure regularly/often (from themselves or others) (0.76)**
	4.	Experiences performance pressure regularly/often (from themselves or others) (0.80)**	Parents/guardians spend a lot of time with the child (0.75)*	Engaging in music, singing, acting, drawing/painting, or photography outside school hours with a club or association: 1 day per week (0.77)*
	5.	Parents/guardians have clear rules about what the child can and cannot do (0.84)*	Total MHI5 score (0.99)**	Parents/guardians spend a lot of time with the child (0.85)*
Smoking/Vaping	1.	Parents/guardians usually/always know where he/she is when not at home (0.48)***	Participate in sports at a club or gym: 4–6 days per week (0.41)*** Every day (0.34)**	Parents/guardians usually/always know where he/she is when not at home (0.48)***
	2.	Participate in sports at a club or gym: 2–3 days per week (0.74)** 4–6 days per week (0.52)*** Every day (0.44)***	Engaging in music, singing, acting, drawing/painting, or photography outside school hours with a club or association: 2–3 days per week (0.41)**	Engaging in music, singing, acting, drawing/painting, or photography outside school hours with a club or association: Less than 1 day per week (0.71)* 1 day per week (0.49)*** 2–3 days per week (0.73)**
	3.	Sport or move in free time without a club or gym: 1 day per week (0.72)** 2–3 days per week (0.70)*** 4–6 days per week (0.58)***	Parents/guardians usually/always know where he/she is when not at home (0.41)***	Participate in sports at a club or gym: 2–3 days per week (0.78)* 4–6 days per week (0.57)*** Every day (0.64)*
	4.	Engaging in music, singing, acting, drawing/painting, or photography outside school hours with a club or association: 1 day per week (0.72)* 2–3 days per week (0.76)*	Sport or move in free time without a club or gym: 4–6 days per week (0.44)*	Sport or move in free time without a club or gym: Less than 1 day per week (0.61)*** 1 day per week (0.64)*** 2–3 days per week (0.71)** 4–6 days per week (0.61)*** Every day (0.68)*
	5.	Experiences performance pressure regularly/often (from themselves or others) (0.75)**	Total MHI5 score (0.97)***	Experiences performance pressure regularly/often (from themselves or others) (0.65)***
Cannabis use	1	Parents/guardians usually/always know where he/she is when not at home (0.48)***	Participate in sports at a club or gym: 4–6 days per week (0.35)**	Parents/guardians usually/always know where he/she is when not at home (0.42)***
	2.	Can turn to someone when having a problem or feeling troubled (0.60)**	Being a girl (0.47)**	Engaging in music, singing, acting, drawing/painting, or photography outside school hours with a club or association: 1 day per week (0.51)*
	3.	Participate in sports at a club or gym: 2–3 days per week (0.62)*** 4–6 days per week (0.56)*** Every day (0.48)***	Parents/guardians usually/always know where he/she is when not at home (0.54)*	Participate in sports at a club or gym: 1 day per week (0.62)* 2–3 days per week (0.69)* 4–6 days per week (0.54)**
	4.	Sport or move in free time without a club or gym: 1 day per week (0.69)* 2–3 days per week (0.63)** 4–6 days per week (0.59)** Every day (0.66)*	Total MHI5 score (0.98)*	Sport or move in free time without a club or gym: 4–6 days per week (0.61)*
	5.	Parents/guardians spend a lot of time with the child (0.66)***	-	Being a girl (0.66)*

Odds ratios (OR) with 95% confidence intervals (CI) were obtained from multivariable logistic regression analyses stratified by country. The table presents all statistically significant factors associated with lower odds of adolescent substance use. Complete regression outputs are provided in Appendix I. *p < 0.05, **p < 0.01, ***p < 0.001.

**Table 6:** Overview of cannabis use in the three countries.

Item	Answer	Netherlands			Belgium			Germany		
		*n*=	%	Group diff.	*n*=	%	Group diff.	*n*=	%	Group diff.
Ever used cannabis***	Yes	872	9.1%	a	188	7.0%	b	760	9.3%	a
Used cannabis in the last 4 weeks	Yes	441	4.6%	a,b	98	3.7%	b	405	4.9%	a
Ever used XlTC, cocaine, magic mushrooms, laughing gas, etc. ***	Yes	247	2.7%	a	92	3.5%	a,b	367	4.5%	b
Used XTC, cocaine, magic mushrooms, laughing gas, etc. in the last 4 weeks***	Yes	69	0.7%	a	0	0.0%	b	136	1.7%	c

Values are absolute numbers (*n*) and percentages (%), calculated on complete cases per country (Netherlands *n* = 9,576; Belgium *n* = 2,682; Germany *n* = 8,203). Only affirmative (“Yes”) responses are shown for dichotomous items; the remaining percentages represent “No” responses. For multi-category items, all relevant answer categories are presented. Asterisks (*, **, ***) indicate statistically significant overall between-country differences based on chi^2^ tests (*p < 0.05, **p < 0.01, ***p < 0.001). Superscripts a, b, c within a row denote significant pairwise differences between countries according to Bonferroni-adjusted *post-hoc* comparisons (α = 0.05); identical letters indicate no significant difference.

Overall, substance use prevalence differed between countries (Tables 1–3, 6). Binge drinking and smoking were most common among Belgian adolescents, while cannabis use was more frequent among Dutch adolescents. In the multivariable analyses, models were stratified by country to explore country-specific associations between individual and environmental factors and substance use outcomes. Differences in odds ratios between countries are therefore not interpreted as statistically different as the focus is on the direction and consistency of associations within each country (Appendix I). To establish whether associations between individual/environmental factors and outcomes (alcohol, smoking/vaping, cannabis use) varied between the counties, interactions terms were tested (Supplementary Table S3). All models were adjusted for gender and grade, which function as demographic background variables.

### Individual and environmental factors

Table 1 summarizes the individual and environmental characteristics of adolescents across the three countries. These factors include demographic and psychosocial variables (e.g., gender, stress, school experience) as well as contextual indicators such as parental monitoring and leisure activities. Several of these characteristics differed between countries. German adolescents reported higher stress levels and lower parental control, whereas Dutch adolescents were most active in organized sports. These background differences provide important context for interpreting the cross-national variations in substance use described above.

With respect to individual factors, adolescents in Germany more frequently reported experiencing (very) high levels of stress (63.6%) compared to those in Belgium (56.5%) and the Netherlands (51.2%) (Table 1). Conversely, Dutch adolescents were more likely to report having someone to turn to when feeling troubled (95.3%) than their peers in Belgium (91.6%) and Germany (89.0%).

Beyond these individual factors, adolescents’ behavior is also shaped by environmental factors such as leisure activities and parental monitoring, which show distinct patterns across the border regions (Table 1). Adolescents in the Dutch border region were more frequently out after 22:00 (17.5%) compared to their peers in the German (15.9%) and Belgian (12.8%) border regions. Parental rule-setting also varied as German parents in the border region were the least likely to set clear rules for their children (62.8%), while Dutch parents enforced rules most often (83.7%), with Belgian parents in between (77.9%). Parental knowledge of adolescents’ whereabouts was generally high across all regions (Netherlands 89.5%, Belgium 92.1%, and Germany 91.4%).

Taken together, these findings highlight some contextual contrasts in family and leisure environments that may relate to the broader social conditions in which adolescent substance-use behaviors develop, although these aspects were not directly examined in this study.

### Alcohol use

Binge drinking (≥5 alcoholic drinks on a single occasion in the past 4 weeks) was comparable across the three border regions (NL 21.7%, BE 22.9%, DE 20.7%) The proportion of adolescents who reported being drunk or tipsy during this period was lower, but showed a similar pattern (NL 17.7%, BE 18.2%, DE 17.1%).

Differences were observed in how adolescents obtain alcohol. Dutch adolescents were less likely to report purchasing alcohol themselves (17.2%) than their peers in Belgium (32.1%) and German (27.1%), whereas receiving alcohol from their parents or other adults was reported more often in the Dutch border region (from parents: NL 49.3% vs. BE 42.1% and DE 46.3%; from other adults: NL 26.8% vs. BE 18.8% and DE 18.7%) (Table 2). Reported parental approval of drinking was higher in the German border region (49.2%) than in the Dutch (38.4%) and Belgian (37.8%) regions, while Dutch adolescents most frequently reported having friends who drink alcohol (NL 67.7%, BE 64.1%, DE 60.4%). Together, these descriptive patterns suggest that social availability (parents/other adults) may be relatively more salient in the Dutch context, whereas commercial availability (self-purchase) features more in Belgium and Germany. A detailed pairwise comparisons are provided in Table 2.

The results show a consistent association between peer context and binge drinking across countries, as adolescents whose friends drink alcohol had substantially higher odds of engaging in binge drinking (NL: OR 9.56, BE: OR 11.23, DE: OR 9.11). Being out of the house for three or more nights per week after 22:00 was also associated with higher odds in each country (NL: OR 2.95, BE: OR 3.43, DE: OR 2.62). Although, interaction analyses indicated that the strength of certain associations, particularly those related to stress, psychological well-being, and late-night outings, varied between countries, the direction of effects remained the same, suggesting that these mechanisms operate similarly across border regions.

Several factors showed protective associations with binge drinking across settings (Table 5). Parental knowledge of adolescents’ whereabouts revealed lower odds in all border regions (NL: OR 0.57, BE: OR 0.58, DE: OR 0.76), as were clear parental rules (NL: OR 0.83, BE: OR 0.75, DE: OR 0.72). Participation in structured leisure activities, such as independent sports or cultural activities (e.g., music, photography), were also associated with lower odds, though the strength of this association varied by activity and country.

### Smoking and vaping

Adolescents in the German border region reported smoking both daily (6.2%) and weekly (9.4%) more often than their peers in the Dutch (2.7 and 4.7% respectively) and Belgian (3.7 and 5.5% respectively) border regions (Table 3).

For vaping, Dutch (6.4%) and German (6.6%) adolescents reported similar daily use, whereas Belgian adolescents vaped less frequently (4.4%). A comparable pattern was observed for weekly vaping, with higher prevalence in both the Dutch (10.5%) and German (11.3%) border regions compared to the Belgian border region (7.8%). When considering smoking and vaping together, German adolescents were somewhat more likely to engage in both behaviors simultaneously (8.6%) than their Dutch (7.3%) and Belgian (6.3%) peers (Table 3).

Regression results (see Tables 4, 5; Appendix I) revealed a broadly similar pattern to binge drinking (Appendix I), with peer influence appearing as the strongest predictor of smoking and vaping in all three countries. Adolescents whose friends drink alcohol have higher odds of smoking or vaping (NL: OR 4.39, BE: OR 7.55, DE: OR 3.90). Late-night socialization also increased the likelihood of smoking and vaping, with those staying out after 22:00 on three or more evenings per week showing higher odds across all regions (NL: OR 3.05, BE: OR 4.45, DE: OR 3.15).

Beyond these environmental factors, individual factors were also related to smoking and vaping. Adolescents who viewed school as pointless reported higher odds of smoking or vaping (NL: OR 1.77, BE: OR 1.50, DE: OR 1.30). In contrast, parental supervision showed a protective association as adolescents whose parents usually or always knew their whereabouts were less likely to smoke or vape (NL: OR 0.48, BE: OR 0.41, DE: OR 0.48). Participation in organized sports and, to a lesser extent, cultural activities, was likewise associated with reduced odds, although the strength of these associations varied somewhat between countries. Interaction analyses confirmed that these differences reflected variation in magnitude rather than direction.

### Cannabis use

Lifetime prevalence of cannabis use was similar in the Dutch (9.1%) and German (9.3%) border regions, but somewhat lower in the Belgian border region (7.0%) (Table 6). A comparable pattern was observed for recent cannabis use in the past 4 weeks, with the Dutch (4.6%) and German (4.9%) adolescents reported similar levels, whereas prevalence was slightly lower among Belgian adolescents (3.7%) (Table 6).

These descriptive findings show that cannabis use is relatively comparable between Dutch and German adolescents, while somewhat less common among Belgian youth. Although cannabis use was less prevalent than alcohol use and smoking or vaping, the regression analyses identified a consistent pattern of social and environmental associations across the three countries.

Regression results (Tables 4, 5; Appendix I) indicated that peer context was associated with cannabis use. Adolescents whose friends drink alcohol had higher odds of cannabis use across all three countries (NL: OR 4.55, BE: OR 6.45, DE: OR 5.31). Late-night socializing was also associated with elevated odds with those staying out three or more nights per week showing (OR between 3.29–3.63 in the three regions). Viewing school as pointless was related to higher odds of cannabis use in the Dutch (OR 1.35) and German (OR 1.45) border regions.

Parental knowledge of adolescents’ whereabouts was consistently associated with lower odds of cannabis use in all border regions (NL: OR 0.48, BE: OR 0.54, DE: OR 0.42). Participation in structured sports was likewise associated with reduced odds of cannabis use in each of the three countries (Tables 4, 5). While interaction analyses showed that the protective effect of parental monitoring differed somewhat in strength between countries, the overall direction of the association was consistent.

## Discussion

This study explored how individual and environmental factors are associated with substance use among adolescents in the border regions of the Netherlands, Belgium and Germany. These findings lead to three key messages. First, social environmental factors, particularly peer influence and frequently being out of the house, emerged as the strongest associations with substance use in all countries. Second, factors such as parental monitoring and participation in structured leisure activities were consistently associated with lower substance use, although levels of exposure to these factors differ between regions. Third, behavioral differences between countries could only partially be explained by national policy differences, suggesting that broader cultural and social contexts, which extend across national borders, play an important role in shaping adolescent behavior in these border-region setting where daily life is not confined to national boundaries. This is supported by interaction analyses showing that, although the strength of some associations differed, the underlying relationships were consistent across countries.

### Binge drinking in border regions

In the border regions, binge drinking rates are a concern in all three countries. Although the legal drinking age differs slightly between the Netherlands (18 years), Belgium (16 years for non-strong alcohol), and Germany (14 years under parental supervision and 16 years for beer and wine), these differences appear to have limited impact on adolescent drinking behavior. This aligns with earlier studies showing that alcohol policy influences consumption but is not the sole determining factor (33). Compared with recent national figures from the Dutch Youth Health Monitor (34), which reported a binge drinking prevalence of 18.2% among adolescents aged 13–16 years, the proportion observed in the present study is slightly higher. This suggests that alcohol use may be relatively more common among adolescents living in the Dutch border region. For Belgium and Germany, direct national comparisons are not available for this age group, but the overall findings point to broadly similar behavioral patterns across the three neighboring regions (1, 2).

Findings from this study show that environmental factors, such as social influences, were associated with higher odds of alcohol use. For example, adolescents are significantly more likely to binge drink if their friends drink alcohol, with an increased likelihood of more than nine times in the Netherlands and Germany, and more than eleven times in Belgium. Other studies also emphasize the importance of peer influence (35). In addition, being outside the home after 22:00 at least three times per week was associated with higher odds of binge drinking in all border regions.

Parental practices appear to occupy a dual position in relation to binge drinking with parental approval reflecting a less restrictive home context and parental monitoring reflecting a more protective one. The descriptive analysis showed that Dutch adolescents relatively often obtain alcohol from their parents (49.3%) or from other adults (26.8%), which may indicate a less restrictive social context for adolescent alcohol access. Previous research in the Netherlands has shown that parents often provide alcohol at home based on the belief that supervised drinking is safer than unsupervised consumption (36). UK research similarly indicates that many parents adopt this strategy because they perceive it as a way to reduce risk, although evidence shows it actually increases alcohol use (37). However, this parental provision of alcohol appears, paradoxically, to increase rather than reduce alcohol use (36). Parental supervision, on the other hand, has been associated with lower levels of later adolescent alcohol use (38), and clear parental rules have also been associated with lower alcohol use (36). In border regions, parents may also navigate overlapping normative and regulatory environments, such as differing legal age limits or cultural attitudes toward adolescent alcohol use. These divergent frameworks may complicate parental decision-making about supervision, rule-setting and alcohol provision, as parents confront norms and regulations that do not align neatly across the neighboring countries. Although the present study could not directly assess cross-context parental dilemmas, border studies emphasize that border regions function as multi-layered social spaces where institutional and cultural expectations intersect, potentially shaping parenting practices in subtle ways (17, 30, 36).

Participation in organized leisure activities also plays a complex role among environmental factors. Although sports are generally healthy, they may promote binge drinking, possibly due to social settings where alcohol consumption is seen as a means of social bonding (39).

Taken together, these findings illustrate how adolescents’ drinking behaviors are embedded in interconnected social and cultural contexts. Peer norms, parental practices, and leisure structures reinforce or buffer one another, creating locally shared expectations around drinking. The belief among some parents that controlled drinking at home prevents risk, the social acceptance of alcohol in sports clubs, and the normative pressure from peers are all part of a wider cultural framework in which drinking is often seen as a socially acceptable aspect of growing up (35–39). This interplay of socialization contexts, family, peers, schools, and leisure, helps to explain why adolescent drinking behaviors remain similar across countries despite different legal restrictions.

In border regions, this interaction may be even more complex, as adolescents and their families are exposed to overlapping cultural norms from neighboring countries. While prevention policies are organized nationally, everyday practices are shaped by these shared social environments. This study shows that around 17% of Dutch adolescents who have ever drunk alcohol purchase alcohol themselves, despite the ban on sales to minors. Although this study did not investigate where adolescents buy alcohol, existing literature indicates that differences in commercial availability, for example through varying legal age limits in neighboring countries, may influence adolescent alcohol use (33).

These findings emphasize that binge drinking among adolescents in border regions is not primarily determined by national policy, but rather by environmental factors such as their immediate social environment, peer influence and the level of parental involvement. In border regions, these environmental influences may be shaped by overlapping social norms from neighboring countries, which could dilute or reinforce national policy efforts.

### Smoking and vaping in border regions

Adolescents in the German border region smoke significantly more often than their peers in the Dutch and Belgian border regions. At the same time, the percentages of daily and weekly vaping are highest in the Netherlands and Germany. These differences occur even though legislation regarding the purchase and use of tobacco and e-cigarettes is virtually identical in the three countries. Moreover, the analysis shows that the main factors associated with smoking and vaping, such as peer influence and parental supervision factors, are consistent across all three countries (Table 5). Compared with recent national figures from the Dutch Youth Health Monitor (34), which reported a daily smoking or vaping prevalence of 7.0% and a weekly prevalence of 11.3% among adolescents aged 13–16 years, the proportions observed in the present study are broadly comparable. This indicates that tobacco and e-cigarette use among adolescents in the Dutch border region is largely in line with national patterns. For Belgium and Germany, directly comparable national data for this age group are not available, but overall findings suggest similar behavioral tendencies across the three neighboring regions (1, 2).

The fact that similar individual and environmental factors can lead to different outcomes suggests that wider social and cultural contexts influence how these mechanisms operate. Research show that social norms and the perceived acceptability of smoking within peer groups strongly influence adolescent behavior (40, 41). Longitudinal and network-based studies further highlight the role of peer selection and socialization processes in reinforcing these patterns (42, 43). When smoking or vaping is seen as ‘normal’, adolescents are more likely to start smoking themselves.

Beyond peer norms, local prevention contexts may also explain the observed differences. As emphasized in prevention frameworks such as the Icelandic Prevention Model (IPM), Communities That Care (CTC), and the European Drug Prevention Quality Standards (EDPQS), environmental factors including parental involvement and structured leisure activities are key to reducing adolescent substance use (9–14). While the IPM has gained international attention, experts from the European Society for Prevention Research (EUSPR) have cautioned that its evidence base outside Iceland remains debated and that transferability to other countries is uncertain (44). Legislation alone therefore appears insufficient. It is the daily local context that is most influential. This is supported by findings linking organized leisure activities, especially cultural or individual ones, to lower substance use (45). Salmon (6) further shows that psychosocial vulnerability and the absence of protective conditions like parental supervision or social support are linked to increased e-cigarettes and substances use.

Taken together, these findings demonstrate that the mechanisms underlying smoking and vaping are largely universal, peer norms, parental monitoring, and leisure engagement act as key determinants across all countries, but their strength and manifestation depend on the surrounding social and cultural environment. In border regions, these contextual influences may be amplified, as adolescents may be exposed to multiple normative frameworks that could shape attitudes toward tobacco and e-cigarette use even under comparable legal conditions. This illustrates how policy homogeneity does not guarantee behavioral uniformity in regions where everyday interactions routinely cross national borders.

### Cannabis use in border regions

Although adolescent cannabis use is higher in the Dutch and German border regions than in the Belgian region, the underlying patterns of associations with both higher and lower cannabis use are broadly similar across the three countries. Compared with national figures from the Dutch Youth Health Monitor (34), which reported that 9.1% of adolescents aged 13–16 years had ever used cannabis and 5.0% had used it in the past 4 weeks, the prevalence found in the present study is very similar. This suggests that cannabis use among adolescents in the Dutch border region does not substantially differ from the national average. For Belgium and Germany, directly comparable national data for this age group are lacking, but European monitoring data indicate broadly similar levels of experimentation among adolescents (1, 2).

These results indicate that while the prevalence patterns are comparable, the way factors associated with both higher and lower odds manifest may differ across social and cultural contexts. As Wu et al. (46) highlights, perceptions of normative behavior around cannabis use vary widely between countries and subgroups and strongly influence actual use. In a border region where adolescents may be exposed to multiple normative frameworks, such contextual differences in cultural acceptance and normalization may play an important role.

The results show that peer influence, a key environmental factor associated with higher odds, is strongly linked to cannabis use. This aligns with previous research identifying peer norms and social bonding as key factors in adolescent substance use (40, 47). Hanafin et al. (48) adds that peer smoking is closely tied to the e-cigarette, highlighting the central role of peer behavior in shaping substance use patterns. This mechanism likely operates similarly across borders, although the perceived normalization of cannabis may vary between countries, influencing the strength of these associations.

Among individual factors associated with higher odds for cannabis use, negative school experiences, such as perceiving school as meaningless, are linked to cannabis use in several border regions. This aligns with Henry and Thornberry (49), who identify school disengagement as a pathway to substance use. When school feels unstructured or lacks meaning, adolescents may seek affirmation or fulfillment elsewhere, including through substance use.

Within the environmental context, parental monitoring and structured leisure activities, such as sports, were associated with lower odds of cannabis use, which supports the relevance of environmental conditions associated emphasized across prevention frameworks such as IPM, CTC, and EDPQS-based approaches. Lac and Crano (50) show that effective parental supervision, knowing where adolescents are and with whom, significantly reduces the likelihood of cannabis use. This monitoring provides both external control and supports the internalization of social norms. Steinberg et al. (51) similarly highlight its protective role, even when peers use substances. Other scholars (52, 53) link regular sports participation to lower cannabis use, as sport provides structure, social bonding and alternatives to risk behavior, illustrating the role of the environmental context in adolescent cannabis use. However, recent longitudinal evidence suggests this relationship may be more complex, with some studies showing no consistent protective effect (54). In border regions, where adolescents encounter multiple social settings and cultural influences, the local context in which these activities take place may be particularly important.

Taken together, these findings show that the determinants of cannabis use are largely universal, peer influence, school engagement, parental monitoring, and leisure structure operate similarly across countries, but the magnitude and expression of these associations depend on the wider social and cultural environment. This suggests that national legislation alone cannot explain cross-country differences. Rather, it is the everyday social context in which adolescents grow up that shapes how factors associated with higher and lower odds of cannabis use operate. In border regions, this social context may be influenced by cultural dynamics that extend beyond a single national setting, reflecting the fluid and relational nature of borders. This interpretation corresponds with perspectives from border studies, which describe borders not merely as fixed administrative lines but as dynamic social institutions that shape everyday interactions and local cultural environments (17). Together, these findings suggest that adolescent substance use in these border regions should be interpreted in relation to socio-cultural cultural contexts that do not necessarily align neatly with national policy boundaries.

### Strengths and limitations

This study offers insights into adolescent substance use in the Dutch, Belgian, and German border regions, with several points that deserve attention. First, we used a convenience sample at school level, resulting in this large sample of adolescents from each border region. School recruitment differed between countries, with all eligible schools invited in the Dutch and German border regions and a sampling procedure used in the Belgian border region. This resulted in a smaller Belgian sample and may have influenced participation rates, sample composition and the comparability of absolute prevalence figures between countries. Nevertheless, the sample supports cross-country comparisons and allows robust analyses. Second, the use of self-reported data may introduce social desirability bias. However, data were collected anonymously via a secure online platform, reducing this risk. Third, by focusing on alcohol, tobacco, and cannabis, the study allows for an in-depth and focused analysis, but the exclusion of other relevant health-related behaviors like mental health problems limits broader insight into the clustering of unhealthy behaviors among adolescents. Fourth, binge drinking was chosen over general alcohol consumption as a more specific indicator of substance use, though this may also overlook more moderate but still risky drinking patterns. Fifth, while the study includes a wide range of individual and environmental factors, other unmeasured influences, like impulsivity or cultural norms, may also shape adolescent behavior. Although the selection of variables was informed by existing literature and prevention frameworks, the relatively large number of tested associations warrants cautious interpretation. Confirmation in future hypothesis-driven studies would further strengthen the evidence for the observed associations and their meaningfulness. Sixth, although this study specifically targeted adolescents in border regions, we did not collect information on individual cross-border mobility or distance to the border. Our analyses therefore reflect differences between countries within border regions rather than the direct effects of crossing borders. Because no comparison group from non-border regions was included, the findings should not be interpreted as evidence of border-specific effects, but as patterns and associations observed within border regions. However, it cannot be excluded that adolescents in these regions are exposed to cross-border differences in policies and cultural norms, which future research should examine more directly. Finally, the cross-sectional design captures adolescent substance use at a single point in time but does not allow for causal conclusions. Observed associations should therefore not be interpreted as causal relationships. Still, the large and diverse sample across three countries ensures robust, generalizable findings that contribute to evidence-based prevention strategies.

Despite these limitations, the study remains a strong and reliable contribution to the understanding of adolescent substance use in border regions.

### Cross-border policy implications and future research

Our findings suggest that adolescent substance use in border regions should not be fully interpreted through national policies alone. In these border areas, adolescents may be simultaneously exposed to multiple systems, which could enable some adolescents to encounter or use more ‘favorable’ policy environment, for example lower age limits or cheaper availability across a nearby border. Such dynamics highlight the need for prevention strategies that look beyond country-specific frameworks and consider the Euregional context. Strengthening protective structures, such as parental involvement and meaningful leisure activities, remains relevant, but sustainable impact is more likely when such efforts are embedded in broader community collaborations. In line with frameworks such as CTC, cross-border community coalitions could be established to bring together schools, public health organizations, youth clubs, and law enforcement from neighboring regions. By sharing data and resources, developing unified prevention campaigns, and organizing community events that engage youth from all sides of the border, these coalitions may provide a consistently supportive environment. Although such initiatives require commitment and resources that are often considered beyond local priorities, they offer a realistic step toward collective prevention in border regions. At a policy level, harmonization of legal age limits, pricing policies, and enforcement remains challenging because such decisions are made nationally. Nevertheless, regional stakeholders could play a role by providing comparative evidence from border regions to national policymakers, thereby supporting a stronger lobby for cross-border policy coordination. This type of evidence-based advocacy may help to reduce inconsistencies that adolescents can exploit by seeking the most ‘favorable’ policy context across borders.

Future research should therefore examine how divergent legislation, pricing and prevention frameworks across borders affect adolescent behavior, and to what extent adolescents actively use these opportunities. Comparative studies between border and non-border regions could clarify whether border-specific factors amplify or reduce risks. In addition, studies are needed to evaluate the effectiveness of interventions explicitly designed for cross-border contexts. Such evidence would inform more targeted and context-sensitive prevention strategies that take into account the particular opportunities and vulnerabilities of border regions. This observation is consistent with research on Dutch-German-Belgian border regions showing that, despite European integration, both institutional arrangements and subtle cultural barriers continue to shape daily life, schooling, and leisure across borders (55).

## Conclusion

This study highlights that adolescent substance use, including alcohol, tobacco and cannabis, remains prevalent across border regions despite differences in national regulations. The findings confirm the importance of individual and environmental factors associated with substance use, with peer influence and late-night socialization showing the strongest associations with substance use. However, parental monitoring and structured leisure activities provide important protective effects. By comparing three neighboring countries, the study shows that the observed patterns of vulnerability and protection are broadly consistent, yet their strength and expression vary according to the social and cultural environments shaped by both national and border-region contexts. The results therefore suggest that policy interventions should move beyond legal restrictions and take into account the broader social context in which adolescents in border regions grow up, particularly where overlapping national environments may shape the conditions under which substance-use behavior develops.

## Data Availability

The raw data supporting the conclusions of this article will be made available by the authors, without undue reservation.
