# Supplementary material for: Associations between individual and environmental factors and adolescent substance use in the border regions of the Netherlands, Belgium, and Germany
Source: Front Public Health. 2026 Sep 8;14:1858516. doi: 10.3389/fpubh.2026.1858516 (PMC13593793; doi:10.3389/fpubh.2026.1858516)
Supplement: Supplementary file 1 [file Supplementary_file_1.docx]

Appendix I – Regression analysis Binge drinking, smoking and/or vaping weekly and cannabis use in the last 4 weeks

| **Item** | **BINGE DRINKING** | | | **SMOKING/VAPING** | | | **CANNABIS USE** | | |
| --- | --- | --- | --- | --- | --- | --- | --- | --- | --- |
|  | **Netherlands** OR [95% CI] | **Belgium** OR [95% CI] | **Germany** OR [95% CI] | **Netherlands** OR [95% CI] | **Belgium** OR [95% CI] | **Germany** OR [95% CI] | **Netherlands** OR [95% CI] | **Belgium** OR [95% CI] | **Germany** OR [95% CI] |
| **INDIVIDUAL FACTORS - DEMOGRAPHIC FACTORS** | | | | | | | | | |
| Being a Girl | 1.088 [0.948, 1.250] | **0.673**  **[0.513, 0.883]**** | 0.895  [0.769, 1.042] | **1.331**  **[1.133, 1.563]***** | 0.740  [0.518, 1.059] | **1.189**  **[1.007, 1.405]*** | **0.658**  **[0.518, 0.835]**** | **0.466**  **[0.273, 0.796]**** | **0.664**  **[0.516, 0.856]**** |
| In 4th grade | **2.617 [2.242, 3.055]***** | **2.261**  **[1.732, 2.952]***** | **3.038**  **[2.581, 3.577]***** | **1.412**  **[1.181, 1.688]***** | 0.989  [0.692, 1.413] | **1.272**  **[1.073, 1.507]**** | **2.453**  **[1.813, 3.321]***** | **1.893**  **[1.084, 3.308]*** | **1.386**  **[1.054, 1.824]*** |
| **INDIVIDUAL FACTORS - PSYCHOSOCIAL FACTORS** | | | | | | | | | |
| Total MHI5 score | 0.998  [0.993, 1.003] | **0.988**  **[0.979, 0.997]**** | **0.993**  **[0.988, 0.998]**** | **0.991**  **[0.986, 0.996]**** | **0.974**  **[0.964, 0.985]***** | **0.985**  **[0.980, 0.990]***** | **0.987**  **[0.980, 0.995]**** | **0.980**  **[0.964, 0.996]*** | **0.985**  **[0.978, 0.993]***** |
| Feels (very) often stressed by one or more of the following factors: school or homework, home situation, own problems, what others think of them, everything they have to do | 1.075 [0.92, 1.256] | 0.978  [0.735, 1.301] | **1.317**  **[1.107, 1.566]**** | **1.433**  **[1.194, 1.720]***** | 1.445  [0.975, 2.142] | **1.230**  **[1.010, 1.498]*** | 1.409  [1.068, 1.857]* | 1.772  [0.973, 3.226] | 1.113  [0.822, 1.506] |
| Can turn to someone when having a problem or feeling troubled | 0.863 [0.654, 1.137] | **1.591**  **[1.041, 2.431]*** | **1.424**  **[1.139, 1.781]**** | 0.957  [0.721, 1.270] | 0.960  [0.602, 1.530] | 1.052  [0.848, 1.306] | **0.599**  **[0.419, 0.858]**** | 1.083  [0.531, 2.206] | 0.914  [0.672, 1.243] |
| Experiences performance pressure regularly/often (from themselves or others) | **0.795**  **[0.673, 0.939]**** | 0.843  [0.609, 1.167] | **0.763**  **[0.64, 0.909]**** | **0.754**  **[0.621, 0.915]**** | 0.837  [0.550, 1.276] | **0.648**  **[0.531, 0.791]***** | 1.065  [0.805, 1.410] | 0.967  [0.526, 1.777] | 0.751  [0.555, 1.015] |
| Experiences performance pressure from parent(s)/guardian(s) | **1.344**  **[1.074, 1.682]*** | 1.102  [0.723, 1.680] | 1.138  [0.898, 1.441] | 1.155  [0.901, 1.480] | 1.014  [0.590, 1.745] | **1.535**  **[1.191, 1.979]**** | 1.102  [0.778, 1.560] | 0.649  [0.296, 1.425] | 1.214  [0.829, 1.777] |
| Experiences performance pressure from teachers/school | 0.927 [0.733, 1.171] | 1.062  [0.690, 1.633] | 1.129  [0.893, 1.429] | 0.928  [0.718, 1.199] | 0.79  [0.445, 1.403] | 0.829  [0.645, 1.065] | 1015  [0.707, 1.459] | 1764  [0.789, 3.941] | 0.862  [0.594, 1.250] |
| Experiences performance pressure from friends | 1.217 [0.95, 1.558] | 1.467  [0.940, 2.290] | 1061  [0.835, 1.347] | 1.184  [0.907, 1.546] | 0.708  [0.388, 1.292] | 1.095  [0.851, 1.409] | 0.693  [0.465, 1.032] | 0.995  [0.438, 2.261] | 1.201  [0.828, 1.744] |
| Experiences performance pressure from coach or trainer in sports | 1.101  [0.811, 1.496] | 0.757  [0.447, 1.279] | 1.048  [0.777, 1.413] | 0.903  [0.635, 1.286] | 0.882  [0.419, 1.856] | 0.658  [0.466, 0.931]* | 0.779  [0.458, 1.325] | 0.785  [0.263, 2.342] | 1.173  [0.744, 1.850] |
| Bullied at school in the last 3 months | **1.336**  **[1.106, 1.614]**** | 0.744  [0.522, 1.060] | **1.240**  **[1.061, 1.449]**** | 1.226  [0.997, 1.506] | 0.658  [0.416, 1.041] | 1.092  [0.924, 1.289] | 1.129  [0.831, 1.533] | 0.665  [0.335, 1.318] | 1.155  [0.899, 1.483] |
| Finds going to school pointless | **1.463**  **[1.257, 1.704]***** | **1.357**  **[1.008, 1.827]*** | **1.325**  **[1.100, 1.597]**** | **1.773**  **[1.506, 2.086]***** | **1.501**  **[1.046, 2.153]*** | **1.302**  **[1.076, 1.576]**** | **1.346**  **[1.060, 1.708]*** | 1.223  [0.713, 2.100] | **1.446**  **[1.100, 1.900]**** |
| School experience in 3 categories: |  |  |  |  |  |  |  |  |  |
| Okay | 1.114  [0.961, 1.290] | 1.047  [0.802, 1.366] | 1.019  [0.870, 1.193] | 1.060  [0.884, 1.270] | 1.418  [0.980, 2.053] | 1.089  [0.912, 1.302] | 1.035  [0.781, 1.372] | 0.964  [0.559, 1.663] | 0.829  [0.626, 1.098] |
| Terrible | 1.07  [0.882, 1.298] | 0.974  [0.685, 1.385] | 1.208  [0.992, 1.470] | 1.286  [1.034, 1.598]* | 1.047  [0.661, 1.656] | 1.221  [0.990, 1.506] | 1.290  [0.934, 1.781] | 0.836  [0.428, 1.63] | 1.002  [0.732, 1.371] |
| **ENVIRONMENTAL FACTORS - PHYSICAL ENVIRONMENT** | | | | | | | | | |
| Child is not at home for 3 days or more after 22:00 | **2.950**  **[2.563, 3.396]***** | **3.427**  **[2.522, 4.656]***** | **2.624**  **[2.233, 3.083]***** | **3.047**  **[2.612, 3.554]***** | **4.445**  **[3.134, 6.305]***** | **3.152**  **[2.674, 3.715]***** | **3.634**  **[2.912, 4.535]***** | **3.291**  **[2.010, 5389]***** | **3.196**  **[2.535, 4.028]***** |
| On how many days of the week do you engage in music, singing, acting, drawing/painting, or photography outside school hours with a club or association? |  |  |  |  |  |  |  |  |  |
| Less than 1 day per week | 0.984  [0.770, 1.258] | 1.409  [0.888, 2.235] | 1.135  [0.871, 1.481] | 0.813  [0.610, 1.084] | 0.738  [0.380, 1.434] | **0.711**  **[0.516, 0.979]*** | 0.991  [0.645, 1.522] | 1.187  [0.463, 3.039] | 0.712  [0.423, 1.199] |
| 1 day per week | 0.877  [0.696, 1.105] | 1.189  [0.771, 1.833] | **0.772**  **[0.621, 0.959]*** | **0.721**  **[0.541, 0.961]*** | 0.924  [0.522, 1.634] | **0.493**  **[0.376, 0.646]***** | 0.845  [0.539, 1.325] | 1.231  [0.522, 2.903] | **0.508**  **[0.328, 0.785]**** |
| 2 or 3 days per week | **0.762**  **[0.615, 0.945]*** | 1.035  [0.721, 1.486] | 0.889  [0.718, 1.102] | **0.757**  **[0.589, 0.973]*** | **0.407**  **[0.227, 0.729]**** | **0.728**  **[0.571, 0.928]*** | 0.904  [0.613, 1.334] | 0.691  [0.302, 1.582] | 0.724  [0.490, 1.068] |
| 4 to 6 days per week | 0.771  [0.559, 1.064] | 1.232  [0.680, 2.231] | 0.896  [0.633, 1.268] | 0.812  [0.565, 1.169] | 0.411  [0.168, 1.005] | 0.915  [0.625, 1.341] | 1.120  [0.659, 1.902] | 0.558  [0.150, 2.082] | 1.153  [0.656, 2.026] |
| Every day | 0.908  [0.695, 1.188] | 1.135  [0.717, 1.796] | 0.666  [0.457, 0.971]* | 0.728  [0.535, 0.991]* | 1.109  [0.638, 1.929] | 0.765  [0.522, 1.119] | 1.210  [0.790, 1.852] | 2.069  [0.996, 4.295] | 1.073  [0.640, 1.800] |
| On how many days of the week do you engage in activities outside school hours with a different kind of club, association, or organization? (Ref: (Almost) never) |  |  |  |  |  |  |  |  |  |
| Less than 1 day per week | 1.171  [0.897, 1.528] | 1.586  [0.996, 2.526] | **1.434**  **[1.099, 1.872]**** | 0.966  [0.709, 1.315] | 0.715  [0.362, 1.414] | 0.983  [0.711, 1.360] | 0.725  [0.449, 1.171] | 0.570  [0.191, 1.704] | 1.102  [0.676, 1.798] |
| 1 day per week | **1.277**  **[1.038, 1.571]*** | 1.239  [0.886, 1.732] | 1.215  [0.963, 1.532] | 0.887  [0.690, 1.141] | 0.983  [0.632, 1.528] | 0.986  [0.753, 1.291] | 0.876  [0.600, 1.278] | 0.543  [0.250, 1.176] | 0.994  [0.651, 1.518] |
| 2 or 3 days per week | **1.246**  **[1.055, 1.473]*** | 0.939  [0.664, 1.328] | **1.349**  **[1.095, 1.661]**** | 1087  [0.893, 1.323] | 0.749  [0.455, 1.232] | 1.105  [0.872, 1.400] | 0.844  [0.621, 1.147] | 0.907  [0.461, 1.783] | 1.325  [0.929, 1.890] |
| 4 to 6 days per week | 1.288  [0.991, 1.675] | 1.100  [0.672, 1.799] | 0.979  [0.715, 1.340] | 1035  [0.747, 1.433] | 0.701  [0.324, 1.514] | 1.146  [0.801, 1.640] | 0.897  [0.551, 1.461] | 0.976  [0.341, 2.794] | 0.987  [0.575, 1.693] |
| Every day | 0.695  [0.405, 1.194] | 1.386  [0.561, 3.424] | 1.590  [0.976, 2.589] | 0.961  [0.525, 1.757] | 1.217  [0.362, 4.086] | 1.287  [0.753, 2.198] | 0.482  [0.184, 1.262] | 0.424  [0.049, 3.679] | 1.596  [0.834, 3.052] |
| How often do you participate in sports at a club or gym? (Ref: (Almost) never) |  |  |  |  |  |  |  |  |  |
| Less than 1 day per week | 1.179  [0.805, 1.727] | 1.193  [0.618, 2.301] | 1.396  [0.971, 2.008] | 0.924  [0.615, 1.390] | 0.697  [0.316, 1.537] | 0.966  [0.669, 1.393] | 0.909  [0.522, 1.584] | 0.728  [0.225, 2.359] | 0.892  [0.498, 1.596] |
| 1 day per week | 1.028  [0.804, 1.315] | 0.918  [0.571, 1.477] | **1.378**  **[1.064, 1.786]*** | 0.837  [0.641, 1.092] | 1.200  [0.705, 2.044] | 0.806  [0.609, 1.066] | **0.649**  **[0.425, 0.990]*** | 1.136  [0.514, 2.511] | **0.617**  **[0.383, 0.993]*** |
| 2 or 3 days per week | **1.359**  **[1.131, 1.633]**** | **1.822**  **[1.292, 2.569]**** | **1.385**  **[1.138, 1.687]**** | **0.742**  **[0.609, 0.905]**** | 0.743  [0.490, 1.129] | **0.778**  **[0.634, 0.954]*** | **0.619**  **[0.462, 0.829]**** | 0.701  [0.378, 1.301] | **0.688**  **[0.501, 0.944]*** |
| 4 to 6 days per week | 1.176  [0.949, 1.457] | 1.263  [0.852, 1.871] | **1.374**  **[1.095, 1.724]**** | **0.523**  **[0.409, 0.670]***** | **0.410**  **[0.242, 0.695]**** | **0.574**  **[0.443, 0.743]***** | **0.564**  **[0.399, 0.798]**** | **0.347**  **[0.155, 0.778]*** | **0.537**  **[0.366, 0.789]**** |
| Every day | 0.982  [0.692, 1.396] | 0.960  [0.549, 1.681] | 1.258  [0.909, 1.742] | **0.439**  **[0.284, 0.676]***** | **0.338**  **[0.154, 0.738]**** | **0.635**  **[0.439, 0.918]*** | **0.483**  **[0.260, 0.898]*** | 0.564  [0.205, 1.551] | 0.622  [0.378, 1.025] |
| How often do you sport or move in your free time without a club or gym? (Ref: (Almost) never) |  |  |  |  |  |  |  |  |  |
| Less than 1 day per week | 0.914  [0.715, 1.170] | 0.950  [0.619, 1.458] | 1.171  [0.918, 1.493] | 0.848  [0.648, 1.111] | 0.795  [0.467, 1.354] | **0.607**  **[0.466, 0.790]***** | 1.093  [0.751, 1.591] | 0.533  [0.223, 1.273] | 0.670  [0.432, 1.039] |
| 1 day per week | 0.921  [0.748, 1.133] | 0.842  [0.575, 1.233] | 1.144  [0.918, 1.426] | **0.720**  **[0.570, 0.910]**** | **0.589**  **[0.359, 0.968]*** | **0.637**  **[0.501, 0.809]***** | **0.691**  **[0.487, 0.981]*** | 0.691  [0.332, 1.436] | 0.910  [0.629, 1.316] |
| 2 or 3 days per week | 0.951  [0.788, 1.147] | 0.744  [0.517, 1.070] | 1.024  [0.834, 1.257] | **0.699**  **[0.568, 0.860]**** | 0.900  [0.578, 1.402] | **0.710**  **[0.574, 0.878]**** | **0.626**  **[0.460, 0.852]**** | 0.886  [0.464, 1.694] | 0.971  [0.700, 1.346] |
| 4 to 6 days per week | **0.641**  **[0.508, 0.809]***** | 0.627  [0.383, 1.026] | 0,776  [0.59, 1.019] | **0.576**  **[0.442, 0.750]***** | **0.442**  **[0.209, 0.932]*** | **0.609**  **[0.453, 0.818]**** | **0.586**  **[0.399, 0.860]**** | 0.689  [0.250, 1.897] | **0.614**  **[0.378, 0.998]*** |
| Every day | **0.706**  **[0.549, 0.909]**** | 1.120  [0.711, 1.766] | 0,911  [0.683, 1.214] | 0.775  [0.589, 1.020] | 1.013  [0.574, 1.787] | **0.684**  **[0.503, 0.930]*** | **0.655**  **[0.434, 0.988]*** | 0.984  [0.434, 2.229] | 1.388  [0.910, 2.117] |
| **ENVIRONMENTAL FACTORS - SOCIAL ENVIRONMENT** | | | | | | | | | |
| Most to (almost) all friends drink alcohol | **9.558**  **[8.332, 10.964]***** | **11.232**  **[8.764, 14.395]***** | **9.106**  **[7.932, 10.455]***** | **4.392**  **[3.714, 5.194]***** | **7.546**  **[5.307, 10.728]***** | **3.899**  **[3.319, 4.579]***** | **4.553**  **[3.492, 5.936]***** | **6.454**  **[3.725, 11.182]***** | **5.308**  **[4.122, 6.835]***** |
| Parents/guardians usually/always know where he/she is when not at home | **0.569**  **[0.469, 0.689]***** | **0.567**  **[0.381, 0.843]**** | **0.756**  **[0.603, 0.946]*** | **0.484**  **[0.399, 0.586]***** | **0.408**  **[0.267, 0.623]***** | **0.481**  **[0.39, 0.594]***** | **0.479**  **[0.368, 0.624]***** | **0.541**  **[0.294, 0.996]*** | **0.415**  **[0.314, 0.548]***** |
| Parents/guardians have clear rules about what the child can and cannot do | **0.836**  **[0.711, 0.982]*** | **0.745**  **[0.566, 0.981]*** | **0.723**  **[0.631, 0.828]***** | 0.84  [0.705, 1.001] | 0.752  [0.535, 1.057] | 0.910  [0.784, 1.056] | 0.818  [0.636, 1.051] | 0.693  [0.422, 1.138] | 0.888  [0.707, 1.115] |
| Parents/guardians spend a lot of time with the child | **0.86**  **[0.742, 0.996]*** | **0.753**  **[0.575, 0.988]*** | **0.846**  **[0.732, 0.978]*** | **0.767**  **[0.653, 0.901]**** | 0.852  [0.607, 1.196] | **0.736**  **[0.630, 0.861]***** | **0.663**  **[0.526, 0.836]**** | 1.091  [0.658, 1.810] | **0.694**  **[0.546, 0.882]**** |
| **ENVIRONMENTAL FACTORS - ECONOMIC ENVIRONMENT** | | | | | | | | | |
| Experience some or significant difficulty managing finances at home | 0.819 [0.625, 1.071] | 1.212  [0.807, 1.821] | 0.904  [0.726, 1.126] | 1.361  [1.041, 1.780]* | 1.979  [1.273, 3.078]** | **1.415**  **[1.150, 1.741]**** | 1.150  [0.784, 1.687] | 1.712  [0.939, 3.122] | 1.106  [0.805, 1.520] |

**Legend.** Odds ratios (OR) with 95% confidence intervals (CI) from multivariable logistic regression analyses of binge drinking, smoking and vaping, and cannabis use in the past four weeks, stratified by country (Netherlands, Belgium, Germany). All independent variables were included simultaneously in each model. *p < 0.05, **p < 0.01, ***p < 0.001 indicate statistical significance.
