# Supplementary material for: Associations between individual and environmental factors and adolescent substance use in the border regions of the Netherlands, Belgium, and Germany
Source: Front Public Health. 2026 Sep 8;14:1858516. doi: 10.3389/fpubh.2026.1858516 (PMC13593793; doi:10.3389/fpubh.2026.1858516)
Supplement: Supplementary file 2 [file Table_3.DOCX]

**Supplementary Table S3**. P-values of interaction terms between country and individual/environmental factors for alcohol use, smoking/vaping, and cannabis use.

|  | **Alcohol** (interaction  p-value) | **Smoking** (interaction  p-value) | **Cannabis** (interaction  p-value) |
| --- | --- | --- | --- |
| Being a girl | 0.483 | 0.769 | 0.406 |
| In 4^th^ grade | 0.297 | 0.054 | 0.050 |
| Experience some or significant difficulty managing finances at home | 0.717 | 0.342 | 0.692 |
| Total MHI5 score | 0.001 | 0.008 | 0.238 |
| Feels (very) often stressed by one or more of the following factors: school or homework, home situation, own problems, what others think of them, everything they have to do | <0.001 | 0.675 | 0.945 |
| Can turn to someone when having a problem or feeling troubled | 0.361 | 0.922 | 0.646 |
| Experiences performance pressure regularly/often (from themselves or others) | <0.001 | 0.216 | 0.977 |
| Experiences performance pressure from parent(s)/guardian(s) | 0.091 | 0.021 | 0.499 |
| Experiences performance pressure from teachers/school | 0.100 | 0.225 | 0.596 |
| Experiences performance pressure from friends | 0.547 | 0.798 | 0.307 |
| Experiences performance pressure from coach or trainer in sports | 0.228 | 0.340 | 0.327 |
| Bullied at school in the last 3 months | 0.001 | 0.365 | 0.328 |
| Finds going to school pointless | 0.456 | 0.296 | 0.179 |
| School experience in 3 categories | 0.234 | 0.810 | 0.799 |
| Most to (almost) all friends drink alcohol | 0.896 | 0.472 | 0.211 |
| Child is not at home for 3 days or more after 22:00 | <0.001 | 0.798 | 0.352 |
| Parents/guardians usually/always know where he/she is when not at home | 0.975 | 0.007 | <0.001 |
| Parents/guardians have clear rules about what the child can and cannot do | 0.923 | 0.124 | 0.492 |
| Parents/guardians spend a lot of time with the child | 0.692 | 0.514 | 0.721 |
| On how many days of the week do you engage in music, singing, acting, drawing/painting, or photography outside school hours with a club or association? | 0.928 | 0.008 | 0.613 |
| On how many days of the week do you engage in activities outside school hours with a different kind of club, association, or organization? | 0.365 | 0.345 | 0.253 |
| How often do you participate in sports at a club or gym? | 0.376 | 0.243 | 0.730 |
| How often do you sport or move in your free time without a club or gym? | 0.396 | 0.015 | 0.480 |
